# Melatonin Attenuates Diabetic Myocardial Microvascular Injury through Activating the AMPK/SIRT1 Signaling Pathway

**DOI:** 10.1155/2021/8882130

**Published:** 2021-05-27

**Authors:** Bin Wang, Jinyu Li, Mi Bao, Runji Chen, Haiyan Li, Binger Lu, Meixin Chen, Danmei Huang, Yanmei Zhang, Fenfei Gao, Ganggang Shi

**Affiliations:** ^1^Department of Pharmacology, Shantou University Medical College, Shantou 515041, China; ^2^Pharmaceutical Laboratory, The First Affiliated Hospital, Shantou University Medical College, Shantou 515041, China; ^3^Drug Clinical Trial Institution, The Second Affiliated Hospital, Shantou University Medical College, Shantou 515041, China; ^4^Department of Pharmacy, The First Affiliated Hospital, Shantou University Medical College, Shantou 515041, China; ^5^Department of Cardiovascular Diseases, The First Affiliated Hospital, Shantou University Medical College, Shantou 515041, China

## Abstract

Cardiac microvascular endothelial cell (CMEC) dysfunction is considered as a major contributor to the cardiovascular complications in diabetes mellitus, with oxidative stress caused by hyperglycemia playing a critical role in the progression of CMEC dysfunction. Melatonin is a kind of hormone well known for its antioxidant properties, which has potential protective effects against diabetes mellitus and its complications. However, the role of melatonin on CMEC dysfunction caused by hyperglycemia and its molecular mechanisms underlying these effects has not been clarified. Herein, we investigate the protective effects of melatonin on high glucose- (HG-) evoked oxidative stress and apoptosis in CMECs and underlying mechanisms. Our results revealed that melatonin ameliorated the injury caused by HG in primary cultured rat CMECs. Injury can be accompanied by reduced reactive oxygen species (ROS) and malondialdehyde (MDA) production, and enhanced superoxide dismutase (SOD) activity. Meanwhile, melatonin treatment significantly inhibited HG-induced CMEC apoptosis. Moreover, melatonin increased the activity of the AMPK/SIRT1 signaling axis in CMECs under HG condition, whereas administration of the AMPK inhibitor compound C or SIRT1 silencing partially abrogated the beneficial effects of melatonin. In streptozotocin- (STZ-) evoked diabetic mice, melatonin notably ameliorated cardiac dysfunction and activated the AMPK/SIRT1 signaling. In conclusion, our findings revealed that melatonin attenuates HG-induced CMEC oxidant stress, apoptosis injury, and STZ-induced cardiac dysfunction through regulating the AMPK/SIRT1 signaling pathway.

## 1. Introduction

Cardiovascular disease is considered as a major cause of morbidity and mortality in patients with diabetes mellitus (DM) [1, 2]. Evidences have been suggested that endothelial dysfunction participates in the progression and development of pathogenesis of DM-related cardiovascular complications [3]. Cardiac microvascular endothelial cells (CMECs) are major component of myocardial tissue and essential in keeping both adjacent cardiomyocytes and coronary microvessels during the regulation of myocardial perfusion and coronary reserves [4]. DM and hyperglycemia can change endothelial cells structure and function, which contribute to CMEC dysfunction [5].

Numerous studies have reported that oxidant stress is caused by high glucose (HG) through intracellular reactive oxygenation species (ROS) overgeneration, which was involved in the progression of DM-related cardiovascular complications [6]. The continuous excessive ROS produced and accumulated can promote oxidation of DNA, proteins, and lipids, which subsequently cause endothelial cell dysfunction and the ensuing apoptosis of endothelial cells by intrinsic death pathway [7]. Moreover, experimental evidence indicates that the dysfunction and apoptosis of CMECs occur before the cardiomyocyte pathology damage during diabetes progression [8]. Thus, CMEC functional impairment is thought to be a vital importance pathophysiological event during diabetic cardiovascular complication. Therefore, the protection of CMEC function could be beneficial in the prevention and treating cardiovascular complications associated with diabetes.

The AMP-activated protein kinase (AMPK) is considered as a crucial cellular energy sensor, which regulates energy metabolism and promotes energy conservation and glucose uptake through modulating various downstream molecule expressions [9]. Thus, AMPK participates in the modulation of multiple cellular processes, such as mitochondrial metabolism, inflammation, differentiation, and apoptosis [10–12]. Silent information regulation T1 (SIRT1) is a nicotinamide adenine dinucleotide- (NAD^+^-) dependent class III histone deacetylase that is participated in the multiple cellular biological progresses [13]. It has reported that activation with SIRT1 played a critical role in alleviating diabetes and diabetic complications via decrease of oxidant stress [14]. Recently, the AMPK/SIRT1 signaling pathway was reported to be critical in HG-induced endothelial cells dysfunction, including oxidant stress and apoptosis injury [15, 16].

Melatonin (N-acetyl-5-methoxytryptamine) is a hormone generated and secreted by the pineal gland that exerts protective effects against all kinds of cardiovascular diseases [17]. Previous reports have shown that it is more likely to develop type 2 diabetes in people with lower melatonin secretion [18]. Numerous studies have demonstrated that melatonin is beneficial for reducing diabetes-evoked cardiac dysfunction [19, 20]. The mechanisms of melatonin alleviated the diabetic mouse cardiac dysfunctions that were associated with the activated AMPK or SIRT1 signaling [21]. CMECs are major components of myocardial tissue; however, it has not been clarified whether AMPK/SIRT1 pathway activation is essential for the protective effects of melatonin on HG-treated CMECs. Thus, our research is purposed to investigate the potential beneficial effects of melatonin against HG-induced rat primary CMEC oxidant stress and apoptosis injury and STZ-induced diabetic mouse cardiac dysfunction. The action of the AMPK/SIRT1 signaling axis under the melatonin protecting effects was also elucidated.

## 2. Materials and Methods

### 2.1. Animal Experiments

All animal procedures were performed in this study cohered with the *Guide for the Care and Use of Laboratory Animals* by the National Academy of Sciences and published by the National Institutes of Health (NIH publication no. 86-23, revised 1996). The animal experimental protocols were approved by the Institutional Animal Care and Use Committee of Shantou University Medical College. Male C57BL/6J mice were purchased from Vital River Laboratory Animal Technology Co. Ltd. (Beijing, China). Animals were housed under a 12/12 h light/dark cycle at 22°C and had free access to water and a standard diet.

### 2.2. Reagents

Melatonin, streptozotocin, 2′,7′-dichlorofluorescein acetyl acetate (DCFH-DA), mannitol, and D-glucose were obtained from Sigma-Aldrich (MO, USA). Compound C and endothelial cell growth supplement (ECGS) were obtained from Merck (MA, USA). Fetal bovine serum (FBS) and Dulbecco's modified Eagle medium (DMEM) were purchased from Gibco Laboratory (NY, USA). Superoxide dismutase (SOD) activity and malondialdehyde (MDA) concentration detection kits were purchased from the Institute of Nanjing Jiancheng Bioengineering Institute (Nanjing, China). Detection kit for Annexin V-FITC/PI staining was obtained from Dojindo Laboratories (Kyushu Island, Japan). JC-1 dye kit was obtained from MedChem Express (NJ, USA). Primary antibodies against AMPK, SIRT1, and p-AMPK (Thr^172^) were purchased from Cell Signaling Technology (MA, USA). Antibodies against *β*-actin, GAPDH, and goat anti-mouse or rabbit secondary antibodies were purchased from Wuhan Boster Biological Technology Co., Ltd. (Wuhan, China). Bcl-2 primary antibody was purchased from Proteintech (Wuhan, China). Small interfering ribonucleic acids (siRNAs) were purchased from Shanghai GenePharma Co. Ltd. (Suzhou, China).

### 2.3. Type 1 Diabetes and Drug Treatment

Type 1 diabetes mouse model was constructed as previously described [5, 20]. Eight-week-old C57BL/6J mice were fasted overnight and administered an intraperitoneally injection of 50 mg/kg streptozotocin (STZ) in sodium citrate buffer (pH 4.5) for 5 consecutive days to induce type 1 diabetes. After the first 2 weeks of diabetes induction (e.g., mice with fasting blood glucose levels > 16.6 mmol/L were confirmed with diabetes), 10-week-old mice were administered with intraperitoneal injections of the vehicle or 10 mg/kg/d melatonin for 10 weeks, as previously described [20]. Melatonin was initially dissolved in absolute ethanol and then diluted in 0.5% ethanol at a final concentration with sterile water. At the end of the melatonin treatment (12 weeks after the first injection of STZ), the mouse hearts were harvested for further experiments.

### 2.4. Echocardiography Measurements

Echocardiography was performed using Vevo LAZR photoacoustic imaging system (Fujifilm Visualsonics, Toronto, ON, Canada) to monitor the cardiac function. Left ventricular fractional shortening (LVFS), left ventricular ejection fraction (LVEF), and left ventricular end-systolic volume (LVESV) were calculated from M-mode echocardiographic images using computer algorithms.

### 2.5. Isolation and Culture of CMECs

1-3 days neonatal Sprague-Dawley (SD) rats were performed in this study. The isolation and culture of rat CMECs were performed as previously described [22]. The CMECs were cultured in normal DMEM (5.5 mM glucose) including 10% (*v*/*v*) FBS, 15 mg/L ECGs, 100 U/mL streptomycin, and 100 U/mL penicillin in an incubator (95% air, 5% CO_2_, and humidified atmosphere) at 37°C. Experiments used CMECs after 3-6 passages for this study. In the normal control group, CMECs were cultured with normal glucose DMEM supplemented with 2% (*v*/*v*) FBS. In the isosmotic control group, CMECs were cultured with the DMEM including 5.5 mM D-glucose and 27.5 mM mannitol. And in the HG group, CMECs were cultured with DMEM (33 mM D-glucose) for 48 h with or without 2 *μ*M melatonin. To estimate the role of AMPK in mediating melatonin's protective effects, CMECs were treated with HG medium containing melatonin with or without 1 *μ*M compound C (an AMPK antagonist) for 48 h.

### 2.6. Detection of ROS Production in CMECs

The intracellular ROS generation was detected using a DCFH-DA probe with flow cytometry as previously described [23]. Briefly, CMECs were washed with phosphate-buffered saline (PBS) and then trypsinized. Subsequently, the CMECs were rinsed with PBS for three times and then once with DMEM without serum. Next, the CMECs were incubated with the final concentration of 5 *μ*M DCFH-DA in a cell cultured incubator at 37°C for 30 min under the dark. After incubation, the CMECs were rinsed again with ice-cold PBS for three times and detected with the BD Accuri™ C6 flow cytometry (BD Biosciences, CA, USA) at 525 nm emission wavelength and a 488 nm excitation wavelength. The BD Accuri™ C6 flow cytometer was used to analyze the mean fluorescent intensity (MFI).

### 2.7. Determination of MDA Concentration and SOD Activity

First, ice PBS was used to wash CMECs for three times. Second, RIPA lysis buffer including proteinase inhibitors was used to lyse CMECs for 30 min on ice. Next, the lysates were centrifuged with 12,000 rp

m at 4°C for 15 min. MDA concentrations and SOD activity in cell lysate were examined following the manufacturer's protocol.

### 2.8. Apoptosis Measurement Using Flow Cytometry

The measurement of apoptosis with Annexin V-FITC/PI apoptosis detection kit is according to the manufacturer's protocol by flow cytometry. First, CMECs were rinsed twice with PBS and the supernatant was discarded. Second, CMECs were digested with trypsin containing 0.02% EDTA and added DMEM containing 10% FBS to terminate the digestion and transferred the cells suspension to the tube. Subsequently, cells were centrifuged for 5 minutes with 1,000 rpm and rinsed with PBS twice. Next, the CMEC pellets were resuspended with Annexin V binding solution. The 100 *μ*L CMEC suspension was added with 5 *μ*L Annexin V/FITC conjugate and 5 *μ*L propidium iodide and incubated at room temperature for 15 min in the dark. The cells were immediately mixed with 400 *μ*L Annexin V binding solution, and the samples were detected with the BD Accuri™ C6 flow cytometer.

### 2.9. Western Blot Analysis

Protein expressions were determined using western blot according to previous description [23]. Briefly, CMECs were lysed with RIPA lysis buffer including protease inhibitor. Next, the protein concentrations were measured using the Pierce™ bicinchoninic acid (BCA) protein assay kit. Equal amounts of protein samples were boiled in sample buffer for 5 min before separation in 10% or 12% SDS-PAGE gels. The proteins were then transferred to a nitrocellulose membrane. After blocking with Tris-buffered saline containing 0.1% Tween 20 (TBST) and 5% nonfat dried milk at room temperature for 60 min, the nitrocellulose membranes were incubated overnight at 4°C with primary antibodies. After the primary antibodies' incubation, the membranes were rinsed with TBST for three times. Then, the membranes were incubated with horseradish peroxidase-conjugated secondary antibodies at room temperature for 1 h. The membranes were rinsed with TBST buffer for three times and every time for 10 min each. Finally, the protein bands were visualized with SuperSignal detection kit. The density of the target protein bands was performed by the Gel-Pro Image Analysis Software. Densitometry of the bands was normalized to that of *β*-actin.

### 2.10. Small Interfering RNA (siRNA) Transfection of Cells

For *SIRT1* silencing, CMECs were transfected siRNA with Lipofectamine™ 2000 reagent (Invitrogen). CMECs were plated to 6 cm culture dishes and transfected siRNA with Gibco™ Opti-MEM™ medium (Life Technologies, USA) when cells achieved 70%-80% confluence. Briefly, 5 *μ*L negative control (NC) or 5 *μ*L *SIRT1* siRNA (20 *μ*M) was mixed with 250 *μ*L Opti-MEM™ medium. Next, Lipofectamine™ 2000 reagent was added to Opti-MEM™ medium, and subsequently, the mixtures were incubated at room temperature for 20 min. And then, the mixtures were added to culture dishes of primary cultured CMECs for 5 h in an incubator (95% air, 5% CO_2_, and humidified atmosphere) at 37°C. The CMECs were cultured with HG DMEM in the presence of 2 *μ*M melatonin for 48 h. Finally, cells were harvested for subsequent experiments. The special *SIRT1* siRNA sequences were sense: 5′-GAU CCU CGA ACA AUU CUU AdTdT-3′, antisense: 5′-UAA GAA UUG UUC GAG GUC dTdT-3′. The special negative control sequences were sense: 5′-CGU UUG UUC GCU UCC UGA GTT-3′, antisense: 5′-CUC AGG AAG CGA ACA AAC GTG-3′.

### 2.11. Detection of Mitochondrial Membrane Potential (Δ*ψm*)

The changes of Δ*ψm* were observed with JC-1 staining dye assay kit. Firstly, CMECs were incubated with 1 *μ*g/mL JC-1 staining for 15 min in the dark at 37°C. Secondly, the CMECs were rinsed with PBS for three times and subsequently observed and imaged using a Zeiss LSM 880 confocal laser microscopy (Oberkochen, Germany). Red fluorescence generation indicated that JC-1 was concentrated in mitochondrial matrix forming aggregates and represented the high Δ*ψm*. In contrast, a green fluorescence signal indicated that the JC-1 retained the monomer in the cytoplasm and represented the low Δ*ψm*. The reflection of the degree of Δ*ψm* is usually presented by the relative percentage of green and red fluorescence. The reduction of the red/green ratio is commonly used as an indication of apoptosis. The intensities of fluorescence were analyzed by ImageJ software.

### 2.12. Statistical Analysis

All data were displayed as the mean ± standard deviation (SD) of at least three independently repeated experiments. The statistical significance of differences was determined by one-way analysis of variance (ANOVA) followed by Bonferroni's multiple comparisons for post hoc *t-*test in GraphPad Prism 5.0 (GraphPad Software, San Diego, CA, USA). *p* < 0.05 was considered as statistical significance.

## 3. Results

### 3.1. Melatonin Ameliorated HG-Induced Oxidative Stress in CMECs

**Figure 1:**
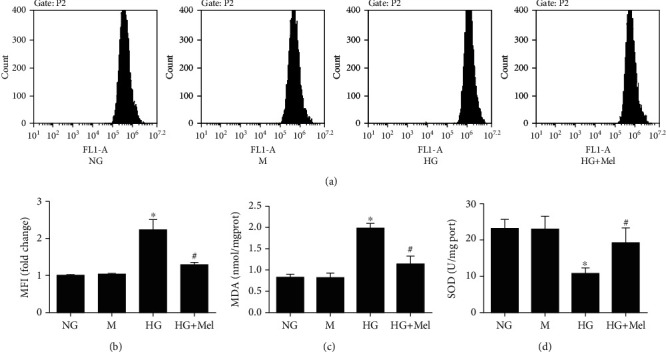
Melatonin prevented oxidant stress induced by HG in CMECs. (a) Flow cytometry to detect ROS production. (b) ROS mean fluorescent intensity. (c) MDA concentrations. (d) SOD activity. Data were expressed as the means ± SD (*n* = 3). ^∗^*p* < 0.05 vs. NG and ^#^*p* < 0.05 vs. HG. NG: normal glucose; HG: high glucose; M: mannitol; Mel: melatonin.

To determine the HG-dependent antioxidant action of melatonin caused in CMECs, we detected some independent parameters, including intracellular ROS generation, MDA content, and SOD activity. Previous evidence suggested that ROS play a critical regulatory role in cardiovascular disease of diabetes [24]. Thus, we detected ROS generation using a DCFH-DA fluorescent probe by flow cytometry in CMECs. Compared to the NG group, intracellular ROS production showed no remarkable changes in the mannitol group. ROS production in CMECs was remarkably increased in the HG group compared with the NG group. However, with melatonin treatment, the increases of intracellular ROS production induced by HG were efficiently reversed (Figures 1(a) and 1(b)). Furthermore, the MDA concentrations and the SOD activity were not significant alterations between the mannitol group and NG group (Figures 1(c) and 1(d)). Compared to the NG group, the MDA concentrations were enhanced and the SOD activity was reduced in HG-cultured CMECs (*p* < 0.05), which indicated that HG provoked oxidant stress in CMECs. On the contrary, these changes of MDA concentrations and SOD activity in HG conditions were dramatically reversed with melatonin treatment. Taken together, these finding suggested that melatonin treatment improved HG-evoked oxidant stress in CMECs in terms of these findings.

### 3.2. Melatonin Attenuated HG-Stimulated Apoptosis in CMECs

**Figure 2:**
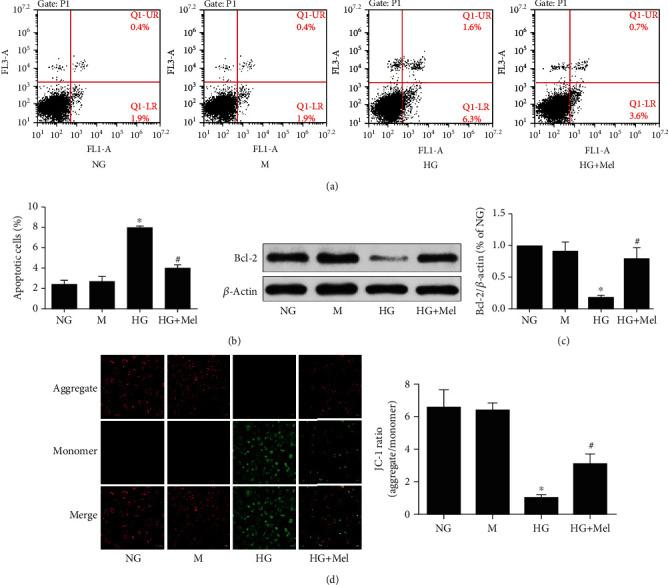
Melatonin attenuated HG-stimulated apoptosis in CMECs. (a) Annexin V-FITC/PI staining to determine apoptosis. (b) Quantification of apoptotic percentage. (c) Western blot to analyze the Bcl-2 expression. (d) Representative pictures of JC-1 staining (magnification, 400x). Data were expressed as the means ± SD (*n* = 3). ^∗^*p* < 0.05 vs. NG and ^#^*p* < 0.05 vs. HG. NG: normal glucose; M: mannitol; HG: high glucose; Mel: melatonin.

Subsequently, we detect the roles of melatonin on HG-evoked apoptosis in CMECs. Annexin V-FITC/PI double staining detection with flow cytometer showed that the apoptotic percentage was remarkably increased in HG conditions (*p* < 0.05) in primary cultured of CMECs (Figures 2(a) and 2(b)). However, melatonin treatment markedly decreased the ratio of apoptotic cells in CMECs exposed to HG (*p* < 0.05), suggesting that melatonin is protected against HG-induced CMEC apoptosis. Furthermore, Bcl-2 expression, an antiapoptotic protein, was also determined by a western blot. In comparison with the NG group, the Bcl-2 protein expression in the HG group significantly decreased; however, this change was reversed when melatonin treatment is in HG-cultured CMECs (Figure 2(c)). By using JC-1 dye staining, we also observed that the green fluorescence was remarkably enhanced as well as the red fluorescence was dramatically reduced in HG-treated CMECs, which produced a decreased ratio of red/green signals and indicated that cell apoptosis had happened. However, treated with melatonin dramatically inhibited HG-stimulated increase in green fluorescence and reduction in red fluorescence (Figure 2(d)). These findings provided direct evidence that melatonin increased mitochondrial membrane potential, ameliorated mitochondrial damage, and decreased apoptosis.

### 3.3. Melatonin Enhanced the Activation of the AMPK/SIRT1 Signaling in HG-Treated CMECs

**Figure 3:**
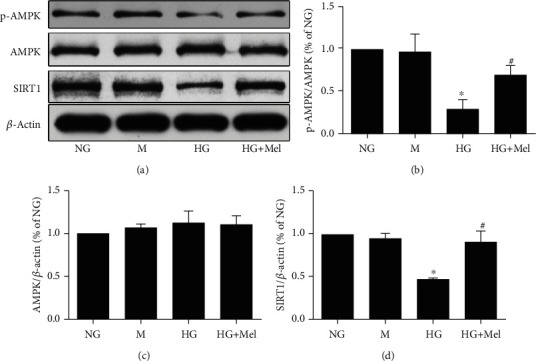
Effects of melatonin on the AMPK/SIRT1 signaling axis in CMECs exposed to HG. (a) p-AMPK Thr^172^, AMPK, and SIRT1 expressions were analyzed with western blot. (b–d) Quantitative analysis of p-AMPK Thr^172^, AMPK, SIRT1 expressions. Data were expressed as the means ± SD (*n* = 3). ^∗^*p* < 0.05 vs. NG and ^#^*p* < 0.05 vs. HG. NG: normal glucose; M: mannitol; HG: high glucose; Mel: melatonin.

Previous evidences have suggested that the AMPK and SIRT1 signaling participate in alleviating diabetes and its correlation complications via reducing oxidant stress and apoptosis injury [25, 26]. To investigate whether the antioxidant and antiapoptotic effects of melatonin are due to modulation of the AMPK and SIRT1 signaling, we examined the AMPK and SIRT1 expressions. Compared to NG conditions, p-AMPK and SIRT1 expressions showed a significant reduction in CMECs exposed to HG conditions, but there were no differences in the total AMPK expression (Figure 3). However, when melatonin was added into HG-cultured CMECs, protein levels of p-AMPK and SIRT1 were substantially elevated when compared to the HG group.

**Figure 4:**
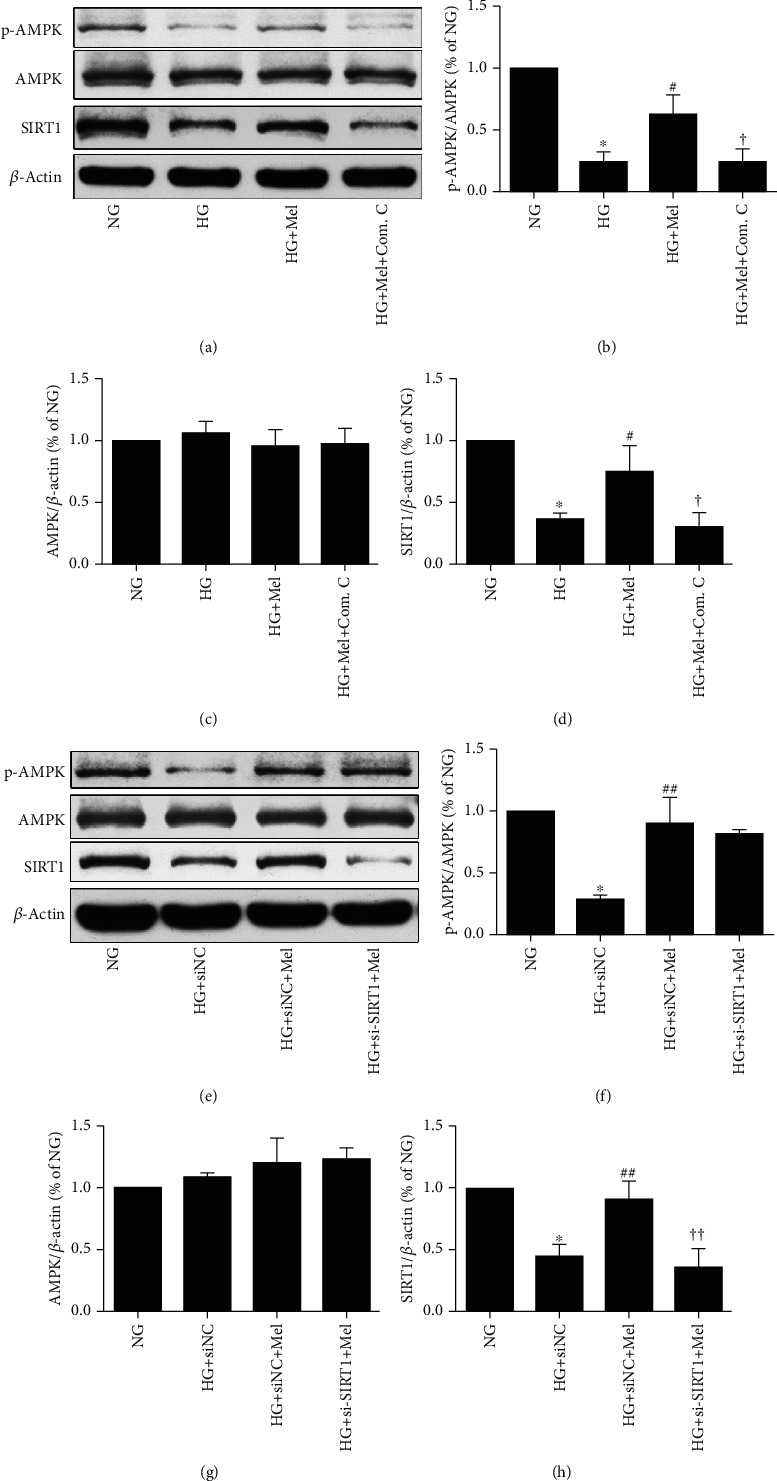
Impacts of compound C and SIRT1 siRNA on the melatonin-stimulated AMPK/SIRT1 signaling in CMECs. (a) p-AMPK Thr^172^, AMPK, and SIRT1 expressions were assessed using western blot in CMECs treated with compound C. (b–d) Quantitative analysis of p-AMPK Thr^172^, AMPK, and SIRT1 expressions. (e) p-AMPK Thr^172^, AMPK, and SIRT1 expressions were determined using western blot in CMEC transfection with SIRT1 siRNA. (f–h) Quantitative analysis of p-AMPK Thr^172^, AMPK, and SIRT1 expressions. Data were expressed as the means ± SD (*n* = 3). ^∗^*p* < 0.05 vs. NG, ^#^*p* < 0.05 vs. HG, ^†^*p* < 0.05 vs. HG+Mel, ^##^*p* < 0.05 vs. HG+siNC, and ^††^*p* < 0.05 vs. HG+siNC+Mel. NG: normal glucose; HG: high glucose; Mel: melatonin; Com. C: compound C; NC: negative control.

Evidence has suggested that AMPK and SIRT1could regulate each other and they shared downstream signaling molecules [27]. Therefore, we examined the relationship of the signaling molecules between AMPK and SIRT1 in CMECs treated with melatonin in HG conditions. As shown in Figure 4, inhibiting the AMPK signaling with compound C notably blunted the melatonin-stimulated activation of AMPK activity and reduced the expression of SIRT1. However, *SIRT1* siRNA significantly reduced the melatonin-induced enhancement of the SIRT1 expression, but it had no significant influence on AMPK activity. Collectively, our data suggested that AMPK might be located upstream of the SIRT1 signaling in the HG environment in melatonin-treated CMECs.

### 3.4. Melatonin Mitigated HG-Induced CMEC Oxidant Stress via Activating the AMPK/SIRT1 Signaling Pathway

**Figure 5:**
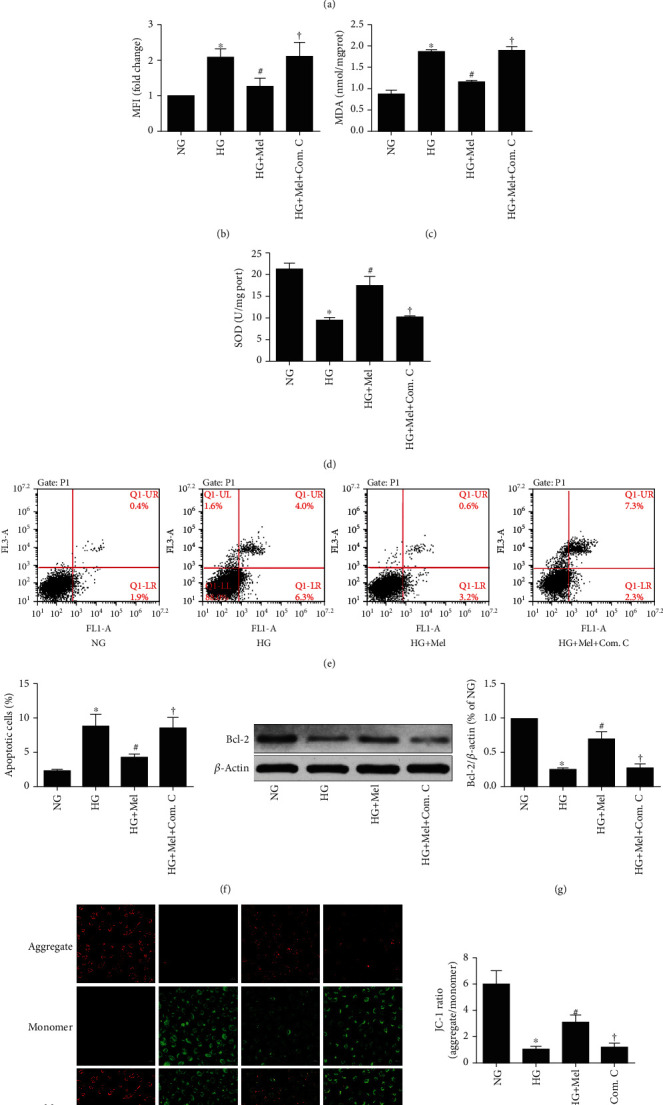
Inhibition of AMPK with compound C abrogated the beneficial effects of melatonin against oxidant stress and apoptosis in HG-cultured CMECs. (a) Flow cytometry analysis of ROS level by DCFH-DA probe. (b) ROS mean fluorescent intensity. (c) MDA concentrations. (d) SOD activity. (e) Apoptosis of Annexin V-FITC/PI to evaluate apoptosis. (f) Quantification histograms indicated the apoptotic percentage. (g) Western blot to estimate Bcl-2 expression. (h) Representative pictures of JC-1 staining (magnification, 400x). Data were expressed as the means ± SD (*n* = 3). ^∗^*p* < 0.05 vs. NG, ^#^*p* < 0.05 vs. HG, and ^†^*p* < 0.05 vs. HG+Mel. NG: normal glucose; HG: high glucose; Mel: melatonin; Com. C: compound C.

**Figure 6:**
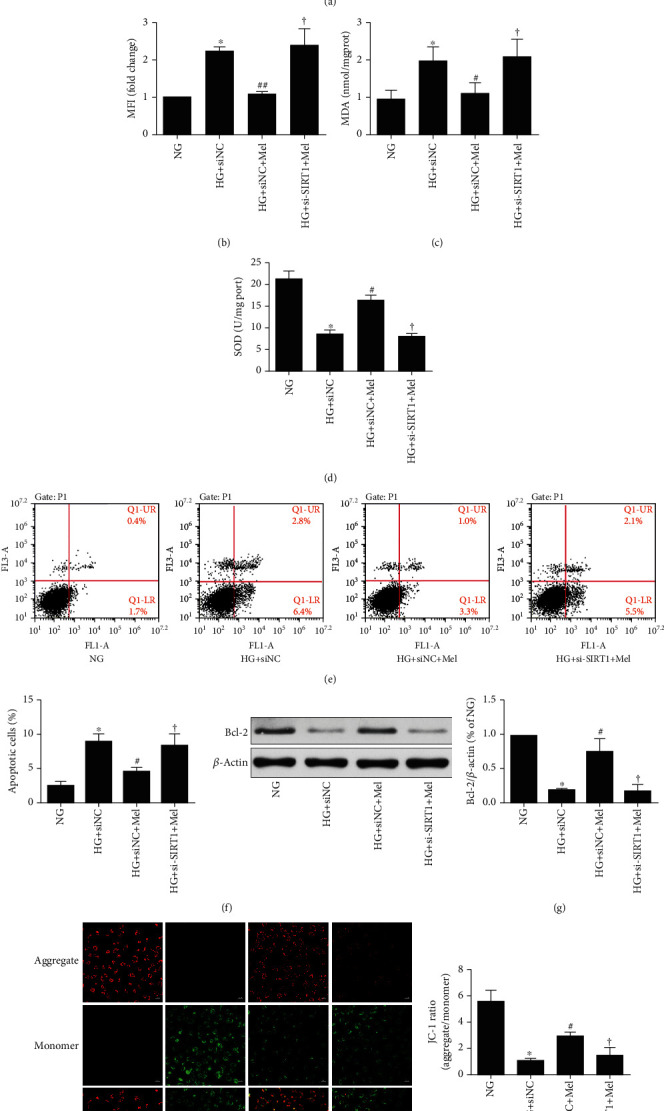
Efforts of melatonin on SIRT1-silenced CMEC oxidant stress and apoptosis induced by HG in CMECs. (a) DCFH-DA to measure ROS. (b) ROS amounts quantified by mean fluorescence intensities. (c) Levels of MDA. (d) Activity of SOD. (e) Annexin V-FITC/PI to evaluate apoptosis extent. (f) Quantification histograms to indicate the apoptotic percentage. (g) Western blot to examine Bcl-2 expression. (h) Representative pictures of JC-1 staining (magnification, 400x). Data were expressed as the means ± SD (*n* = 3).^∗^*p* < 0.05 vs. NG, ^#^*p* < 0.05 vs. HG+siNC, and ^†^*p* < 0.05 vs. HG+siNC+Mel. NG: normal glucose; HG: high glucose; Mel: melatonin; NC: negative control.

To further ascertain whether the AMPK/SIRT1 signaling axis is participated in melatonin-mediated beneficial effects against HG-evoked oxidant damage, the AMPK special inhibitor compound C and transfection with *SIRT1* siRNA were used in this study. As shown in Figures 5(a)–5(d) and Figures 6(a)–6(d), treatment with melatonin remarkably inhibited ROS generation and MDA content and increased SOD activity, whereas either compound C or *SIRT1* siRNA partly abrogated the antioxidative effects. These results indicated that melatonin reduced the cellular oxidant stress damage that was induced by hyperglycemia through enhancing the AMPK/SIRT1 signaling in CMECs.

### 3.5. Melatonin Reduced HG-Evoked CMEC Apoptosis through the AMPK/SIRT1 Signaling Axis

We next investigated whether the AMPK/SIRT1 signaling was involved in melatonin-mediated protection against HG-evoked apoptosis damage. Flow cytometry detection showed that the antiapoptotic effects of melatonin remarkably blunted by compound C or *SIRT1* siRNA (Figures 5(e) and 5(f) and Figures 6(e) and 6(f)). Additionally, compound C or *SIRT1* siRNA abolished the effects of melatonin on the increase of Bcl-2 expression and mitochondrial membrane potential in HG-treated CMECs (Figures 5(g) and 5(h) and Figures 6(g) and 6(h)). These data demonstrated that inhibiting the AMPK/SIRT1 signaling axis significantly abolished melatonin's antiapoptotic effects.

### 3.6. Melatonin Ameliorated Diabetes-Induced Cardiac Dysfunction In Vivo

**Figure 7:**
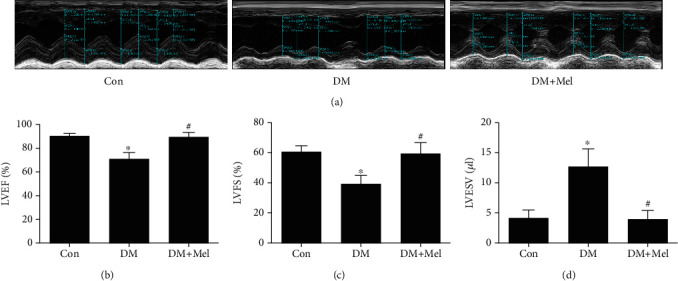
Melatonin alleviated cardiac dysfunction in diabetic mice. (a) Representative images of echocardiography. (b) Left ventricular ejection fraction (LVEF). (c) Left ventricular fractional shortening (LVFS). (d) Left ventricular en-systolic volume (LVESV). Data were expressed as the means ± SD (*n* = 3‐8).^∗^*p* < 0.05 vs. Con and ^#^*p* < 0.05 vs. DM. Con: control; DM: diabetes mellitus; Mel: melatonin.

Echocardiography was used to estimate the cardiac function of diabetic mice. As shown in Figure 7, diabetic mice developed cardiac dysfunction as evidenced by reduced LVEF and LVFS, as well as increased LVESV compared to control mice. However, melatonin treatment significantly improved cardiac function, including increased LVEF and LVFS and decreased LVESV compared with diabetic mice.

### 3.7. Melatonin Improved Diabetic Cardiac Function by Activating the AMPK/SIRT1 Signaling

**Figure 8:**
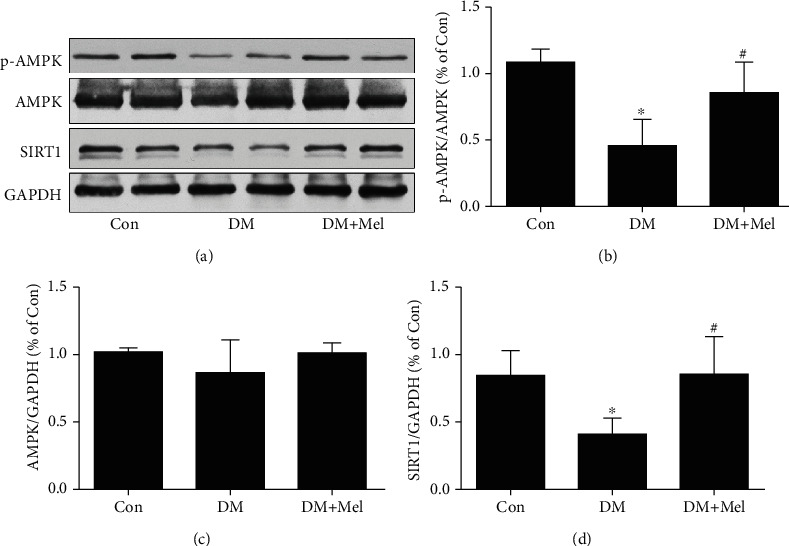
The melatonin-activated AMPK/SIRT1 signaling pathway in myocardial tissues of diabetic mice. (a) p-AMPK Thr^172^, AMPK, and SIRT1 expressions were analyzed with western blot. (b–d) Quantitative analysis of p-AMPK Thr^172^, AMPK, and SIRT1 expressions. Data were expressed as the means ± SD (*n* = 3‐8).^∗^*p* < 0.05 vs. Con and ^#^*p* < 0.05 vs. DM. Con: control; DM: diabetes mellitus; Mel: melatonin.

To further confirm the mechanisms of improving cardiac function in diabetic mice, we measured the activity of AMPK and the expression of SIRT1 using western blot analysis. As shown in Figure 8, the expression of p-AMPK and SIRT1 remarkably decreased in diabetic mice compared to control mice. Interestingly, melatonin treatment notably enhanced p-AMPK and SIRT1 expressions in diabetic mice. Collectively, our data suggested that melatonin ameliorated diabetic cardiac dysfunction via activating the AMPK/SIRT1 signaling pathway.

## 4. Discussion

It is well known that melatonin exerts a strong activity against DM and its related complications [28, 29]. In the current investigation, we observed the actions of melatonin on hyperglycemia-triggered oxidative stress and apoptosis injury in primary cultured rat CMECs *in vitro* and STZ-induced cardiac dysfunction in diabetic mice *in vivo*. We found that melatonin can significantly protect CMECs against HG-induced cellular oxidant stress and apoptosis injury, as well as improve cardiac function in diabetic mice. Our results revealed that the protective effects of melatonin at least partly enhance the AMPK/SIRT1 signaling axis activities.

Numerous evidences have indicated that oxidant stress plays an essential role in DM-associated cardiovascular complication progression and is often correlated with CMEC dysfunction [30]. Previous studies have been showed that hyperglycemia could induce oxidant stress injury in mammal cells, such as mouse microvascular endothelial cells [31], cardiomyocyte [32], and HK-2 cells [33]. Consistent with findings of these other studies, our results showed that hyperglycemia enhanced intracellular ROS and MDA production, and inhibited SOD activity in CMECs. It has been reported that melatonin possessed strong antioxidant and scavenging free radical properties [34]. Tiong et al. also demonstrated that melatonin inhibited HG-induced oxidative stress injury by maintaining the mitochondria integrity [35]. In this study, the HG-induced oxidant stress injury in CMECs was significantly reversed by melatonin. Studies showed that hyperglycemia results in overproduction and accumulation of ROS in mitochondria, which triggered endogenous apoptotic signaling that eventually leads to cell death [16]. We also observed that hyperglycemia visibly enhanced the ratio of apoptotic cells as well as significantly reduced the Δ*ψm* and Bcl-2 protein expressions in CMECs. However, the changes that were induced by hyperglycemia were reversed by melatonin. These data indicated that melatonin exerts the protective effects against CMEC oxidative stress and apoptosis injury that is caused by hyperglycemia.

Accumulated data have shown that AMPK is a prospective molecular target for attenuating HG-induced injury because of its critical actions in modulating apoptosis and oxidative stress [36]. Mitochondria are the main sources of ROS, and mitochondrial dysfunction and damage caused by HG lead to ROS overproduction and accumulation, which provoked the occurrence of cellular oxidative stress and apoptosis [37]. It has been reported that HG induced endothelial mitochondrial dysfunction in an AMPK-dependent manner [38]. Zhang et al. have demonstrated that melatonin could attenuate ischemia/reperfusion-induced mitochondrial dysfunction through activating the AMPK signaling [39]. Liu and colleagues [40] reported that enhancement of the activities of AMPK by melatonin could suppress doxorubicin-evoked myocardial damage through ameliorating mitochondrial oxidant stress and apoptosis. Meanwhile, Zhou et al. also demonstrated that melatonin alleviated ischemia/reperfusion-induced CMEC injury via activation of AMPK [41]. Consistently with these studies, we showed that the p-AMPK expression was reduced in HG conditions in CMECs, while these changes were remarkably reversed by melatonin to confirm that melatonin attenuated HG-induced CMEC oxidant and apoptosis injury via AMPK activation, and AMPK-specific inhibitor was administered with melatonin. The oxidant stress and apoptosis injury caused by hyperglycemia were both significantly inhibited by melatonin; however, these beneficial effects were partly reduced by the inhibition of the AMPK signaling. These results indicate that AMPK mediates the protective effects of melatonin.

Accumulating evidence has shown that sirtuins are key modulators of diabetes and diabetic complications [42]. SIRT1 is the first member of sirtuins family and has been extensively investigated because of its critical protective effects against cardiovascular diseases [43]. Moreover, many investigators have reported that SIRT1 was a promising target to abate various diabetic complications because it regulates apoptosis and oxidative stress. Zhang et al. reported that enhancing SIRT1 protein expression could attenuate the type 1 diabetic rat myocardial ischemia/reperfusion injuries through inhibiting apoptosis and oxidative stress [44]. Arunachalam et al. also reported that a classic antidiabetic drug metformin could attenuate oxidant stress, apoptosis, and senescence in hyperglycemia-stimulated mouse microvascular endothelial cells through activation of the SIRT1 signaling pathway [31]. Besides, several studies have reported that the enhancement of SIRT1 expression after melatonin treatment could alleviate diabetes-associated cardiovascular complications by reducing apoptosis and oxidant stress [20, 45]. In the current study, we also observed that the SIRT1 expression was reduced in CMECs exposed to HG environments. However, treatment with melatonin could reverse this reduction in SIRT1 expression in HG-treated CMECs. To further confirm that the beneficial effects of melatonin are related to SIRT1 expression, we knocked down the SIRT1 expression using siRNA. We found that SIRT1 silencing reduced the positive effects of melatonin against apoptosis and oxidant stress in CMECs exposed to HG conditions. As is known to all, SIRT1 and AMPK could modulate each other. It has been reported that overexpression SIRT1 could increase AMPK activity by enhancing the expression of liver kinase B-1 (LKB1, an AMPK upstream kinase) [46]. Moreover, AMPK positively potentiates SIRT1 expression because of increasing levels of NAD^+^ [47]. Recently, numerous investigators have demonstrated that the activated AMPK/SIRT1 signaling axis was a potential mechanistic target for inhibiting oxidant stress and apoptosis in diabetic models [48, 49]. Indeed, our findings were consistent with these studies, further indicating that AMPK inhibitor compound C could inhibit melatonin induced by the enhancement of SIRT1 expression, while *SIRT1* siRNA had no influence on AMPK activity. Therefore, melatonin might augment the SIRT1 signaling via AMPK activation. Collectively, we demonstrated that melatonin could attenuate hyperglycemia-stimulated oxidant stress and apoptosis damage in CMECs via an AMPK/SIRT1 signaling dependent pathway.

CMECs are the primarily components of cardiac microvasculature that occur earlier pathological damage compared with cardiomyocytes in myocardial tissue during diabetes progression or myocardial ischemia/reperfusion injury [4, 50]. Reduction of CMEC damage could alleviate cardiac dysfunction in diabetic mice, and therefore, the protection of CMECs is an essential therapeutic strategy for diabetes-associated cardiovascular diseases [5]. Consistently with these studies, our results also revealed that melatonin treatment notably improved cardiac function and increased the AMPK/SIRT1 signaling axis activities. Alleviation of the dysfunction of CMECs by melatonin may be involved in protecting against diabetes-induced cardiac dysfunction.

## 5. Conclusion

Overall, our results provide convincing evidence that melatonin can suppress HG-triggered apoptosis and cellular oxidative injury in CMECs and STZ-induced cardiac dysfunction in diabetic mice. These effects are due to enhance the AMPK/SIRT1 signaling pathway activities, which are one of the molecular mechanisms that participate in the melatonin's protective effects. The current research offers insight into melatonin as a potential medication for treating diabetic cardiovascular complications through reducing CMEC dysfunction.

## Data Availability

The data used to support the findings of this study are available from the corresponding author upon request.
