# Corrigendum to “Melatonin Attenuates Diabetic Myocardial Microvascular Injury through Activating the AMPK/SIRT1 Signaling Pathway”

**DOI:** 10.1155/2021/9793010

**Published:** 2021-12-11

**Authors:** Bin Wang, Jinyu Li, Mi Bao, Runji Chen, Haiyan Li, Binger Lu, Meixin Chen, Danmei Huang, Yanmei Zhang, Fenfei Gao, Ganggang Shi

**Affiliations:** ^1^Department of Pharmacology, Shantou University Medical College, Shantou 515041, China; ^2^Pharmaceutical Laboratory, The First Affiliated Hospital, Shantou University Medical College, Shantou 515041, China; ^3^Drug Clinical Trial Institution, The Second Affiliated Hospital, Shantou University Medical College, Shantou 515041, China; ^4^Department of Pharmacy, The First Affiliated Hospital, Shantou University Medical College, Shantou 515041, China; ^5^Department of Cardiovascular Diseases, The First Affiliated Hospital, Shantou University Medical College, Shantou 515041, China

In the article titled “Melatonin Attenuates Diabetic Myocardial Microvascular Injury through Activating the AMPK/SIRT1 Signaling Pathway” [1], there are errors in Figures 2, 5, and 6 due to the incorrect selection of images during manuscript preparation. The authors apologize for these errors and confirm that it does not affect the results and the conclusions of the article. The corrected figures, as approved by the editorial board, are as follows:

## Figures and Tables

**Figure 1:**
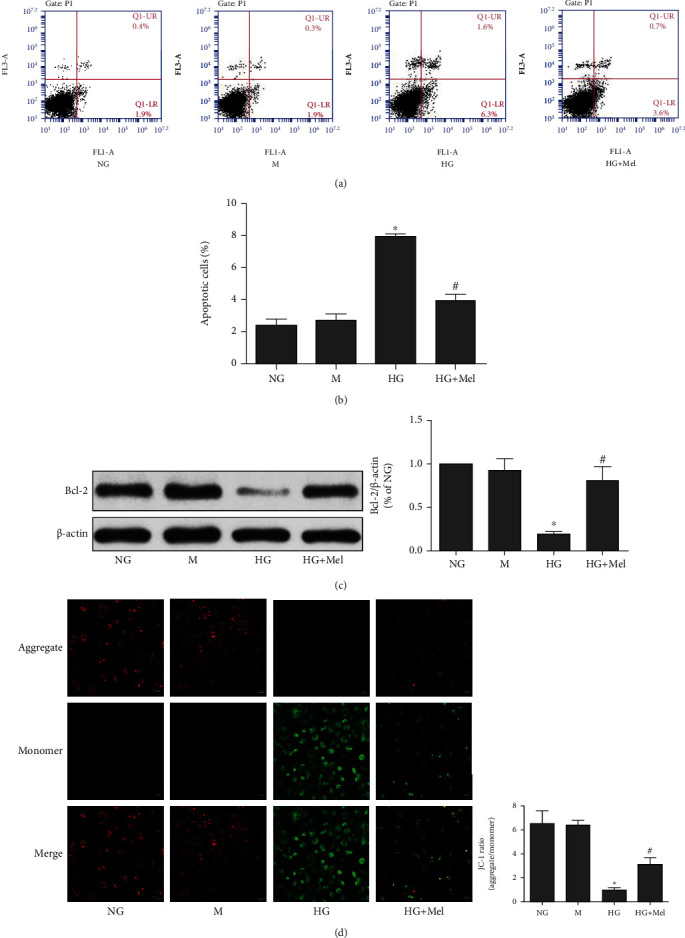
Melatonin attenuated HG-stimulated apoptosis in CMECs. (a) Annexin V-FITC/PI staining to determine apoptosis. (b) Quantification of apoptotic percentage. (c) Western blot to analyze the Bcl-2 expression. (d) Representative pictures of JC-1 staining (magnification 400x). Data were expressed as means ± SD (*n* = 3). ^∗^*p* < 0.05 vs. NG, ^#^*p* < 0.05 vs. HG. NG: normal glucose; M: mannitol; HG: high glucose; Mel: melatonin.

**Figure 2:**
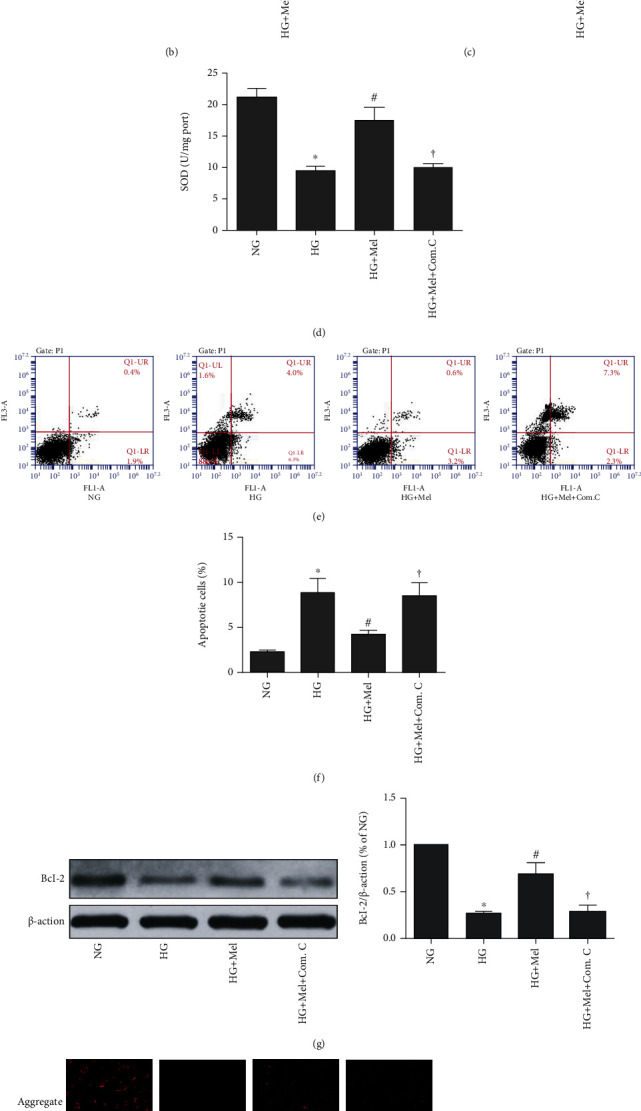
Inhibition AMPK with compound C abrogated the beneficial effects of melatonin against oxidant stress and apoptosis in HG cultured CMECs. (a) Flow cytometry analysis of ROS level by DCFH-DA probe. (b) ROS mean fluorescent intensity. (c) MDA concentrations. (d) SOD activity. (e) Apoptosis of Annexin V-FITC/PI to evaluate apoptosis. (f) Quantification histograms indicated the apoptotic percentage. (g) Western blot to estimate Bcl-2 expression. (h) Representative pictures of JC-1 staining (magnification 400x). Data were expressed as means ± SD (*n* = 3). ^∗^*p* < 0.05 vs. NG, ^#^*p* < 0.05 vs. HG, ^†^*p* < 0.05 vs. HG+Mel. NG: normal glucose; HG: high glucose; Mel: melatonin; Com. C: compound C.

**Figure 3:**
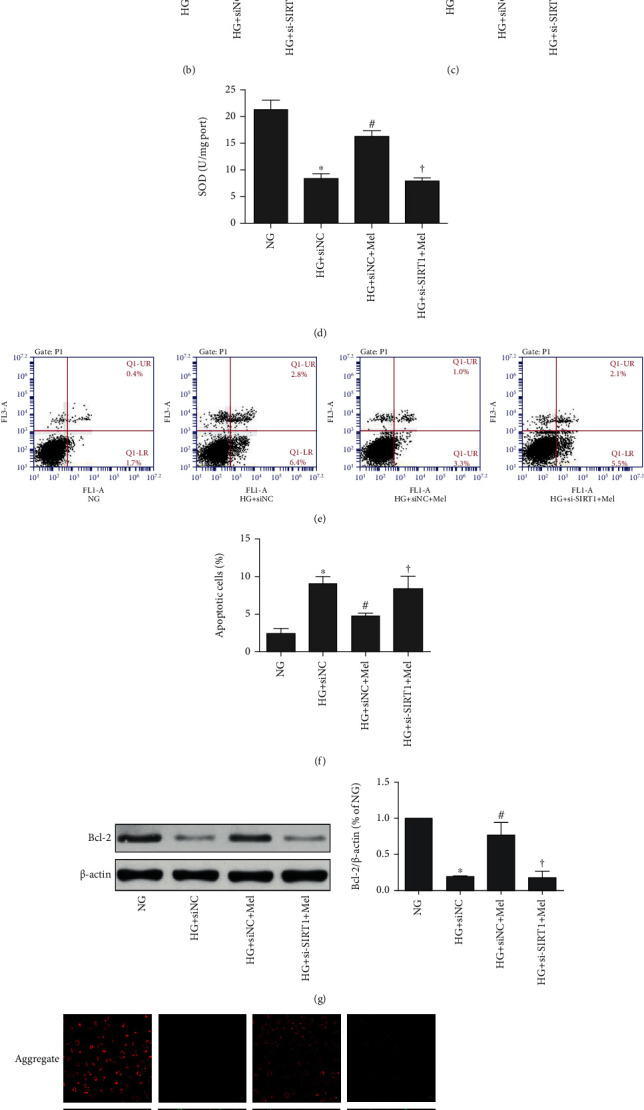
Efforts of melatonin on SIRT1-silenced CMEC oxidant stress and apoptosis induced by HG in CMECs. (a) DCFH-DA to measure ROS. (b) ROS amounts quantified by mean fluorescence intensities. (c) Levels of MDA. (d) Activity of SOD. (e) Annexin V-FITC/PI to evaluate apoptosis extent. (f) Quantification histograms to indicate the apoptotic percentage. (g) Western blot to examine Bcl-2 expression. (h) Representative pictures of JC-1 staining (magnification 400x). Data were expressed as means ± SD (*n* = 3). ^∗^*p* < 0.05 vs. NG, ^#^*p* < 0.05 vs. HG+siNC, and ^†^*p* < 0.05 vs. HG+siNC+Mel. NG: normal glucose; HG: high glucose; Mel: melatonin; NC: negative control.
